# Genomic selection for non-key traits in radiata pine when the documented pedigree is corrected using DNA marker information

**DOI:** 10.1186/s12864-019-6420-8

**Published:** 2019-12-27

**Authors:** Yongjun Li, Jaroslav Klápště, Emily Telfer, Phillip Wilcox, Natalie Graham, Lucy Macdonald, Heidi S. Dungey

**Affiliations:** 1 Scion (New Zealand Forest Research Institute), Private Bag 3020, Rotorua, 3046 New Zealand; 2 Agriclture Victoria, AgriBio Centre, 5 Ring Road, Bundoora, VIC 3083 Australia; 3 University of Otago, 362 Leith Steet, North Dunedin, Dunedin, 9016 New Zealand

**Keywords:** Genomic selection, Non-key traits, Radiata pine, Pedigree correction, Accuracy, Predictive ability

## Abstract

**Background:**

Non-key traits (NKTs) in radiata pine (*Pinus radiata* D. Don) refer to traits other than growth, wood density and stiffness, but still of interest to breeders. Branch-cluster frequency, stem straightness, external resin bleeding and internal checking are examples of such traits and are targeted for improvement in radiata pine research programmes. Genomic selection can be conducted before the performance of selection candidates is available so that generation intervals can be reduced. Radiata pine is a species with a long generation interval, which if reduced could significantly increase genetic gain per unit of time. The aim of this study was to evaluate the accuracy and predictive ability of genomic selection and its efficiency over traditional forward selection in radiata pine for the following NKTs: branch-cluster frequency, stem straightness, internal checking, and external resin bleeding.

**Results:**

Nine hundred and eighty-eight individuals were genotyped using exome capture genotyping by sequencing (GBS) and 67,168 single nucleotide polymorphisms (SNPs) used to develop genomic estimated breeding values (GEBVs) with genomic best linear unbiased prediction (GBLUP). The documented pedigree was corrected using a subset of 704 SNPs. The percentage of trio parentage confirmed was about 49% and about 50% of parents were re-assigned. The accuracy of GEBVs was 0.55–0.75 when using the documented pedigree and 0.61–0.80 when using the SNP-corrected pedigree. A higher percentage of additive genetic variance was explained and a higher predictive ability was observed when using the SNP-corrected pedigree than using the documented pedigree. With the documented pedigree, genomic selection was similar to traditional forward selection when assuming a generation interval of 17 years, but worse than traditional forward selection when assuming a generation interval of 14 years. After the pedigree was corrected, genomic selection led to 37–115% and 13–77% additional genetic gain over traditional forward selection when generation intervals of 17 years and 14 years were assumed, respectively.

**Conclusion:**

It was concluded that genomic selection with a pedigree corrected by SNP information was an efficient way of improving non-key traits in radiata pine breeding.

## Introduction

Genomic selection (GS) is an approach for improving quantitative traits in forest tree breeding populations that uses high density markers dispersed across the whole genome [1–7]. Genomic predictions are estimated based on information from markers, phenotypes and pedigrees to increase the accuracy of breeding values. There are two groups of individuals that are used in genomic selection: the training individuals and the selection candidates. Marker and pedigree information is available for both groups of individuals, but phenotypes are only available for the training individuals. The breeding values of selection candidates can be estimated without the need to ascertain their own individual phenotypes. In traditional tree breeding, selection candidates must be tested in field trials over a number of years to obtain their performance measurements. With genomic selection, breeding cycles can skip the field performance testing phase thereby significantly reducing the generation interval. This benefit of genomic selection is particularly important to species with long generation intervals and requiring large field testing experiments such as forest trees [1, 5, 8] and is particularly useful for those traits that express late in life (e.g. wood density) or have low to medium heritability (e.g. growth and disease resistance) [9, 10]. Until recently, obtaining sufficient single nucleotide polymorphisms (SNPs) to cover the entire genome and hence capture enough genomic variation was prohibitively expensive. The development of next-generation sequencing techniques has enabled researchers to obtain tens of thousands of SNPs at a reasonable cost through genotyping-by-sequencing (GBS) [11]. GBS uses methylation-sensitive restriction enzymes to reduce genome complexity and avoid the repetitive fraction of the genomes. It is becoming increasingly important to acquire genomic information in plant species with complex genomes that lack reference genomes. Where expressed sequence data are available, exome-capture GBS offers an alternative that allows researchers to focus on gene regions, generating a smaller, more manageable dataset and a cost-effective sequencing solution for studying genomes in species with large genomes, such as loblolly pine (*Pinus taeda*) [12].

SNPs have been found to be associated with phenotypic performance [13–15]. Traits under selection can follow specific genetic architectures so several models assuming different distributions of marker effects should be investigated. There are essentially two types of genetic architecture: (1) genetic effects follow a mixed inheritance process where there are few genetic variants of large effects and many variants of very small effects, or (2) genetic effects follow Fisher’s infinitesimal model and each effect contributes only a very small fraction of the total genetic variance. Variable selection procedures such as Bayesian methodologies (including BayesB, BayesC, BayesCπ, etc.) are successively used in traits with the first type of genetic architecture, where the marker effects are modeled to follow a priori distributions [16–18]. These Bayesian methodologies for genomic selection are implemented in two steps: 1) breeding values are estimated using phenotypes and pedigree information, and 2) prediction equations using SNP markers are estimated using de-regressed estimated breeding values (EBVs) as inputs, and then used to derive genomic EBVs (GEBVs) [19–21]. The genomic breeding values of selection candidates are calculated based on the prediction equations and their marker genotypes. However, this two-step procedure has been found to inflate the accuracy of genetic evaluation when individuals with only small numbers of offspring were used [22]. The second type of genetic architecture can be successively fitted by genomic best linear unbiased prediction (GBLUP) which estimates genomic breeding values by incorporating genomic relationships derived from markers in a mixed model framework. No prediction equations are estimated for individual markers. The GBLUP method is preferred for forest tree breeding programmes since only shallow and simple pedigrees are usually available, so reliable de-regression of EBVs cannot be undertaken [23]. Moreover, experimental design features can be included in the model. The genotype by environment interaction can also be formulated and variance-covariance structures incorporated into GBLUP models to account for genetic/residual heterogeneity [3].

Growth, wood density and stiffness are the most economically important traits for radiata pine (*Pinus radiata* D. Don) growers and the improvement of these traits has been the main focus of radiata pine breeding programmes. They are called the key traits (KTs) in radiata pine breeding, while other traits of interest to radiata pine breeders are called non-key traits (NKTs) [24–26]. Branch-cluster frequency, stem straightness, external resin bleeding, and internal checking are examples of such traits. These non-key traits have been targeted for improvement in previous radiata pine research programmes [27, 28]. Selection indices have been proposed to incorporate non-key traits together with the key traits into breeding programmes in New Zealand [25, 26].

New Zealand’s Radiata Pine Breeding Company (RPBC) has established a genomic selection project as part of its overall goal of genetically improving the growth, form, wood quality, and resistance to pests and diseases of radiata pine. Phenotypes for two form traits (branch-cluster and stem straightness) and two wood quality traits (internal checking and external resin bleeding) were available for the training population of this genomic selection project. Branch-cluster frequency refers to the frequency of branch-clusters between one and six metres above the ground on the main stem. It affects both branch size and mean internode length, particularly in the first 3–11 m of the tree bole above the ground. Stem straightness affects log grade, log length and sawn-timber recovery [25, 29]. External resin bleeding and internal checking are two wood defects in radiata pine timber and lower the value of appearance-grade timber, leading to large economic losses for the forest industry [28]. Stem straightness and branch-cluster frequency both have medium to high heritabilities [30] while external resin bleeding and internal checking have low to high heritabilities [26, 31, 32].

This study was the application of genomic selection in radiata pine breeding with a limited number of genotypes in the training population. The objective of this study was to demonstrate the efficacy of applying genomic selection in radiata pine breeding for the non-key traits described. The accuracy of genomic breeding values, the predictive ability of genomic selection, and the expected genetic gains for these non-key traits in radiata pine were investigated in this study.

## Results

### Pedigree correction

The training population in this study comprised two clonally propagated radiata pine breeding trial series planted in New Zealand: POP2 and POP3. Trio parentage assignments for POP2 and POP3 was conducted with Cervus [33, 34]. The percentage of trio parentage confirmed was 48.91 and 49.33% for POP2 and POP3, respectively. There were 83 parents in total in the documented pedigree of POP2 and POP3. About 50% of parents were re-assigned in the SNP-corrected pedigree. The total number of parents in the SNP-corrected pedigree was 107.

### Heritability and accuracy of breeding values

The heritability estimates (${h}_a^2$) in ABLUP (best linear unbiased prediction using the average numerator relationship matrix) and the combined heritability estimates (${h}_{am}^2$) in GBLUP were lower when the SNP-corrected pedigree was used compared with the documented pedigree for branch-cluster frequency and stem straightness (Table 1). However, the heritability estimates were similar when comparing the SNP-corrected pedigree and the documented pedigree for internal checking and external resin bleeding. In GBLUP, the marker-based heritability **(**${h}_m^2$) was higher when using the SNP-corrected pedigree than using the documented pedigree. The combined heritability estimated in GBLUP was higher than the heritability estimated in ABLUP for branch-cluster frequency, stem straightness and internal checking, whereas heritability estimates for external resin bleeding were similar in GBLUP and ABLUP.

**Table 1:** Heritabilities, accuracy of EBVs and GEBVs, and the percentage of genetic variation explained by SNP markers (%VA) for branch-cluster frequency, stem straightness, internal checking and external resin bleeding when using documented or SNP-corrected pedigrees

Statistical model	Pedigree	Genetic parameter	Branch-cluster frequency	Stem straightness	Internal checking	External resin bleeding
ABLUP	Documented	${h}_a^2$	0.17	0.13	0.23	0.33
		*r*_*EBV*_	0.77	0.73	0.78	0.84
	SNP-corrected	${h}_a^2$	0.09	0.10	0.23	0.34
		*r*_*EBV*_	0.73	0.73	0.77	0.87
GBLUP	Documented	${h}_m^2$	0.18	0.09	0.11	0.12
		${h}_{am}^2$	0.28	0.18	0.29	0.35
		*r*_*GEBV*_	0.75	0.68	0.55	0.56
		%VA	64%	54%	39%	36%
	SNP-corrected	${h}_m^2$	0.21	0.1	0.16	0.18
		${h}_{am}^2$	0.22	0.13	0.28	0.34
		*r*_*GEBV*_	0.80	0.70	0.65	0.61
		%VA	96%	74%	59%	46%

${h}_a^2$: heritability from ABLUP, ${h}_m^2$: marker-based heritability from GBLUP, ${h}_{am}^2$: the combined heritability based on variance explained by SNP markers and residual additive genetic variance from GBLUP. Heritabilities and residual variances reported here are the average across seven sites for branch-cluster frequency and stem straightness, and across four sites for internal checking and external resin bleeding

The accuracy of GEBVs was lower (0.55–0.75) for branch**-**cluster frequency, stem straightness, internal checking and external resin bleeding) than EBVs from ABLUP (0.73–0.84) when using the documented pedigree. The accuracy of GEBVs was higher for branch**-**cluster frequency (0.80) than that of EBVs from ABLUP (0.73) while that of GEBVs for stem straightness, internal checking and external resin bleeding (0.61–0.70) was lower than that of EBVs from ABLUP (0.73–0.87) when using the SNP-corrected pedigree. Higher accuracy was observed for external resin bleeding in ABLUP when using the documented pedigree than using the SNP-corrected pedigree. Similar accuracy was observed in ABLUP when using the documented pedigree and SNP-corrected pedigree for stem straightness and internal checking. Lower accuracy in ABLUP was observed for branch**-**cluster frequency when using the documented pedigree compared with the SNP-corrected pedigree. Lower accuracy was observed in GBLUP when using the documented pedigree than when using the SNP-corrected pedigree for all traits.

For branch**-**cluster frequency and stem straightness, the percentage of additive genetic variance explained by SNP markers was 54–64% in GBLUP using the documented pedigree, and 74–96% in GBLUP using the SNP-corrected pedigree. For internal checking and external resin bleeding, the percentage of additive genetic variance explained by SNP markers was 36–39% in GBLUP using the documented pedigree and 46–59% in GBLUP using the SNP-corrected pedigree.

### Predictive ability of genomic selection

The predictive ability, defined as the average correlation between GEBVs from GBLUP in the cross-validation and EBVs from ABLUP using all phenotypes, increased for branch**-**cluster frequency, stem straightness, internal checking and external resin bleeding when using the SNP-corrected pedigree over the documented pedigree (Table 2). The predictive ability of genomic selection ranged from 0.47 to 0.54 for the four traits examined when using the documented pedigree and ranged from 0.55 to 0.70 when using the SNP-corrected pedigree. The predictive ability of traditional BLUP was higher than that from genomic selection, ranging from 0.65 to 0.77 for the four traits examined when using the documented pedigree, and ranged from 0.64 to 0.78 when using the SNP-corrected pedigree.

**Table 2:** The predictive abilities of genomic selection (${r}_{IH_g}$) and traditional selection (${r}_{IH_a}$) and the relative efficiency (*E*_17_ or *E*_14_) of genomic selection over traditional BLUP selection for branch-cluster frequency, stem straightness, internal checking and external resin bleeding

Trait	Documented pedigree				SNP-corrected pedigree			
	${r}_{IH_g}$	${r}_{IH_a}$	*E*_17_^§^	*E*_14_^†^	${r}_{IH_g}$	${r}_{IH_a}$	*E*_17_	*E*_14_
Branch-cluster frequency	0.50	0.77	1.28	1.05	0.70	0.78	2.15	1.77
Stem straightness	0.47	0.73	0.98	0.81	0.55	0.72	1.51	1.25
Internal checking	0.51	0.71	0.92	0.75	0.59	0.70	1.37	1.13
External resin bleeding	0.54	0.65	0.91	0.75	0.57	0.64	1.02	0.84

^**§**^ *L*_*a*_ = 17 years, ^**†**^ *L*_*a*_ = 14 years

When using the documented pedigree, genomic selection was only superior to the traditional BLUP selection for branch-cluster frequency, and only reached 91–98% of the efficiency of traditional forward selection, with no clonal archive establishment, for the other traits. When using forward selection with the establishment of a clonal archive, the generation interval reduced from 17 years to 14 years and the efficiency of genomic selection was reduced. Genomic selection reached 75–81% of the efficiency of forward selection for stem straightness, internal checking and external resin bleeding, and had similar efficiency to forward selection for branch-cluster frequency.

However, when the pedigree was corrected using SNP information, the efficiency of genomic selection over forward selection increased. When forward selection with a generation interval of 17 years was used, genomic selection was equivalent to forward selection for external resin bleeding but led to 37–115% additional benefit over forward selection for branch-cluster frequency, stem straightness and internal checking. When forward selection with a generation interval of 14 years was used, genomic selection only reached 84% of the efficiency of forward selection for external resin bleeding, but still obtained 13–77% extra genetic gain for branch-cluster frequency, stem straightness and internal checking.

## Discussion

Genomic selection has been conducted for growth and wood properties in *Eucalyptus* [5], white spruce (*Picea glauca* (Moench) Voss) [6, 7], interior spruce (*Picea engelmannii* x *glauca*) [1, 23], and loblolly pine (*Pinus taeda* L.) [2]. Isik et al. [3] conducted genomic selection on growth and stem sweep in maritime pine (*Pinus pinaster* Ait.). For *Eucalyptus*, the accuracy of GEBVs across sites was 0.66–0.79 for growth traits and 0.65–0.88 for wood specific gravity within site [5]. For loblolly pine, the accuracy of genomic breeding values across four sites was 0.65–0.75 for diameter at breast height (DBH) and 0.63–0.74 for height [2]. The current study added two form traits and two wood defect traits that were evaluated for genomic selection in radiata pine. The predictive ability of genomic selection in cross-validation was 0.47–0.50 for branch-cluster frequency and stem straightness in the current study. A similar predictive ability (0.49) of genomic selection was reported for stem sweep in maritime pine [3]. Predictive abilities reported for growth traits were 0.46–0.55 in *Eucalyptus* [2] and 0.43–0.47 in maritime pine [3]. In white spruce, the predictive ability was 0.32–0.44 for wood and growth traits when both training and validation datasets shared individuals of the same families but decreased to 0.13–0.28 when training and validation datasets were made up of individuals from different families [6].

When a SNP-corrected pedigree was used, the predictive ability was quite high (0.55–0.70) and seemed overestimated for branch-cluster frequency and stem straightness, given low heritabilities (0.13–0.22) reported in this paper. The low heritability might be because a narrow-sense heritability rather than a broad-sense heritability was reported. Another reason for the high predictive ability for these two traits could be because the EBV was estimated using a pedigree that was corrected by 704 markers. These 704 markers were a well-selected subset of whole genomic markers that were used in the GBLUP method to estimate GEBV.

Genomic selection can increase the amount of genetic gain per year that is delivered to the forest by shortening the breeding cycle. In the current study, the selection efficiency of genomic selection was 37–115% higher than traditional forward selection when the breeding cycle was reduced from 17 years to 9 years for branch-cluster frequency, stem straightness and internal checking. This is very similar to the efficiency of genomic selection reported in loblolly pine, where the selection efficiency per unit time in genomic selection was 53–112% higher than selection through phenotypes, assuming a reduction of 50% in the breeding cycle [2]. A higher selection efficiency of genomic selection was reported in interior spruce with an increase of 106–133%, assuming a 25% reduction in the breeding cycle [23]. In *Eucalyptus*, the efficiency of genomic selection over traditional selection was 50–100% for a reduction of 50% in the breeding cycle and 200–300% for a reduction of 75% in the breeding cycle [2]. However, simulations of a conifer breeding programme, with a training population size of 2000 and assuming a reduction in the breeding cycle from 17 years to 9 years, demonstrated additional genetic gain from genomic selection was 40% for a trait with low heritability and 95% for a trait with high heritability [18].

The best linear unbiased prediction (BLUP) methodology has been widely applied in livestock and plant breeding programmes to rank selection candidates [35]. It employs an average numerator relationship matrix, derived from the pedigree and based on expected relatedness between individuals, and incorporated in the mixed linear model equations [36]. Correct pedigree information is essential for accurately selecting the right individuals as parents of the next generation. However, pedigree errors are common in breeding programmes for both livestock and plant species, with an average of 10% error reported [37–41]. In the current study, the SNP-corrected pedigree re-assigned half of the documented parents, suggesting parentage error was around 50% in the training population. This pedigree error seemed high compared with that reported in the livestock and crop programmes mentioned above. Both error and missing genomic data could be contributing to the high parentage re-assignment we observed. The genotyping error rate in the exome capture GBS data was estimated to be approximately 5%, based on replicated samples. The rate of missing genotypes in the data used for this parentage reconstruction was about 8%. Additional errors may also have been introduced by human operation throughout the whole process, from pollination to planting in the forest, and sample to collection to DNA extraction and genotyping.

Pedigree errors resulted in incorrect estimates of variance components and heritabilities and decreased breeding value accuracies. The genetic gains of breeding populations could be reduced by 4.3–17% when using incorrect pedigree information [37, 42, 43]. In the current study, the SNP-corrected pedigree considerably increased the accuracy of genomic selection, similar to that reported by Muñoz et al. [44]. The SNP-corrected pedigree also increased the percentage of variation explained by SNP markers from 36 to 64% to 46–96%, which suggests that it is the pedigree correction that increases the benefit of genomic selection over traditional BLUP selection.

Three types of narrow-sense heritabilities of branch-cluster frequency, stem straightness, internal checking and external resin bleeding were estimated using a model assuming homogeneous genetic variance and heterogeneous residual variances across sites. ${\hat{h}}_a^2$ was a pedigree-based heritability estimated through a BLUP model (ABLUP) that used the average numerator relationship calculated from pedigree. ${\hat{h}}_m^2$ was a marker-based heritability estimated thought a genomic BLUP (GBLUP) model that used genomic relationship matrix calculated from genomic data, which indicated a ratio of the additive genetic variation explained by genomic markers. ${\hat{h}}_{am}^2$ was a heritability estimated fitted both genomic relationship matrix and the average numerator relationship matrix simultaneously, which indicated a ratio of the additive genetic variation explained by genomic markers and the residual additive genetic variation that was not explained by genomic markers. Genomic selection was not quite efficient for capturing the additive genetic variations for all these NKT, only explaining 36–64% of total additive genetic variations. After correcting pedigree with the 704 parentage reconstruction markers, genomic selection captured most of the total additive genetic variation for branch-cluster frequency and stem straightness, however, it was not quite efficient for internal checking and external resin bleeding. Therefore, it is important for some traits to fit the residual polygenic genetic effects to capture the residual additive genetic variance when conducting genomic selection.

Narrow-sense heritabilities in the training population ranged from 0.09 to 0.28 for branch-cluster frequency and from 0.10 to 0.18 for straightness in the current study, where data from two trial series were combined in one analysis. Similar narrow-sense heritabilities within each trial series of the training population were reported by Li et al. [45], ranging from 0.13 to 0.28 for branch-cluster frequency and from 0.04 to 0.18 for stem straightness. The heritabilities for these two traits were also within the range reported in the literature. Heritability of branch-cluster frequency in radiata pine has been estimated as 0.19 in control-pollinated populations [46] and 0.37 in juvenile clones [47]. The heritability of stem straightness in radiata pine has been estimated as 0.11 to 0.17 in control-pollinated populations [30, 48, 49] and 0.28 for juvenile clones [47].

For radiata pine, low to moderate heritabilities were reported for external resin bleeding, and low to high heritabilities reported for internal checking in the literature. The narrow-sense heritability was 0.33 for external resin bleeding at a single site, and 0.40 for internal checking across two sites, in an open-pollinated progeny test of 224 first-generation families [31]. In a control-pollinated trial series with 150–165 pollen parents crossed to five Female Testers, the narrow-sense heritability was 0.16 for internal checking across two sites [31]. In another study, heritability for internal checking was 0.04–0.61 with an average of 0.35 at nine sites in six trial series [32].

The training population used in this study was limited both in terms of population size and available phenotypes, with only 988 clonally replicated genotypes available for branch-cluster frequency and stem straightness, and 465 for internal checking and external resin bleeding. Nevertheless, we found that the combination of a SNP-corrected pedigree and GBLUP resulted in accuracies that were acceptable (0.61–0.80). This is a very encouraging result for a population of this size. Accuracies of genomic selection are related to the size of the training population available and simulations suggest that higher accuracies can be achieved with larger training populations [10]. The accuracies of GEBVs for internal checking and external resin bleeding, for which less than half the individuals were phenotyped, were lower than that for branch-cluster frequency and stem straightness (Table 2). Increasing the number of genotypes tested for internal checking and external resin bleeding should increase the accuracy of their GEBVs. The accuracy of GEBVs will likely increase in the future as additional genotypes and phenotypes become available for an expanded training population.

The genotypes used in this study were tested in multiple environments (sites). The genetic model used assumed homogeneous genetic variance and heterogeneous residual variances across different environments. No genotype by environment interaction was considered in this study. A low level of genotype by environment interaction has been previously reported for internal checking, branch-cluster and stem straightness [31, 32, 50]. Li et al. [45] found there were considerable genotype by environment interactions for branch-cluster frequency and stem straightness in both the POP2 and POP3 populations. Models that assumed heterogeneous genetic and residual variances, including factor analytic models [51**],** were also attempted but were unstable and would not converge.

Nevertheless, the accuracy and predictive ability of GEBVs for the traits investigated in this study are promising for the RPBC stakeholders, with potential applications for accelerating breeding of radiata pine. In the future, genomic selection will also be available for testing on additional traits, including key traits and resistance to diseases.

## Conclusion

This study presents the first GEBVs for four non-key traits in the New Zealand radiata pine breeding programme, with a theoretical accuracy of 0.61–0.80 and a predictive ability of 0.55–0.70 for the traits examined, when using a pedigree corrected by SNP marker information. The predictive ability reported for the non-key traits in this study indicates that GEBVs are able to achieve an accuracy of 0.55–0.70 when used to predict individuals that are not included in the training population but have relatedness in common with the training population. These results are encouraging and indicate the method will be effective for operational implementation for these traits in radiata pine improvement. The results from this study appeared to favour the forward selection genomics approach, which will significantly reduce the generation interval of radiata pine. This has the potential to deliver benefits over forward selection of 13–77% or 37–115% for branch-cluster frequency, stem straightness and internal checking, with or without clonal archive establishment, respectively.

## Materials and methods

### Genetic material

Genetic material used in this study was provided by RPBC and data were collected from two RPBC clonally propagated radiata pine breeding trial series planted in New Zealand. Planting of the genetic material and collection of the data complied with the RPBC genetic material planting and data collection guidelines. Details of these two trial series are described by Li et al. [45], where the former was called POP2 and the latter POP3. The first trial series POP2 comprised 457 progeny from 63 parents and were planted in 1997 at two sites (Tarawera and Woodhill forests), with a single-paired mating design. The second trial series POP3 comprised 524 progeny from 24 parents and was planted in 1999 at three sites (Kinleith, Tarawera and Woodhill forests) with a factorial mating design. Tarawera and Kinleith forests located in the central North Island. Woodhill forest locates in the northwest of the North Island. The effective population size was 30.07 based on the status number for the training population [52, 53].

### Phenotypic data

Branch-cluster frequency and stem straightness were assessed at age seven in POP3 and at age 8 in POP2. Branch-cluster frequency was assessed using a 9-point system where 1 = uninodal and 9 = extremely multinodal [49]. Stem straightness was also assessed using a 9-point subjective scale where 1 = crooked and 9 = very straight [48]. Internal checking was assessed as a visual score on a scale of 0–3 in POP3, where 0 = none, 1 = low, 2 = moderate, and 3 = severe. Equivalent visual scores for internal checking in POP2 were obtained by converting the percentage of collapse in increment-cores at breast height, assessed at age 9; 0 = below 3.5%, 1 = 3.5–4.5%; 2 = 4.5–6.5%; and 3 = greater than 6.5% [32]. The severity of external resin bleeding from bark split was assessed at age 9 in the POP2 trial series on a scale of 0–3, where 0 = none, 1 = low, 2 = moderate, and 3 = severe. Although these phenotypes were assessed as categorical traits, the distribution of their scores was close to a normal distribution. A summary of branch-cluster frequency, stem straightness, internal checking, and external resin bleeding data is presented in Table 3.

**Table 3:** Summary of statistics for NKTs in POP2 and POP3

Trial	Trait	N	Mean	SD	Min	Max
POP2	Branch-cluster frequency	5290	6.52	1.49	1	9
	Stem straightness	5289	6.68	1.40	1	9
	Internal checking	2732	1.26	0.99	0	3
	External resin bleeding	2275	0.89	0.87	0	3
POP3	Branch-cluster frequency	6851	4.64	1.87	1	9
	Stem straightness	6851	6.51	1.68	1	9

### Genomic data

Four-hundred and sixty-five progeny from POP2, 523 progeny from POP3, and 117 unrelated individuals from the wider radiata pine breeding population (includ**i**ng 53 parents of POP2 and 24 parents of POP3) were genotyped using the exome capture genotyping by sequencing (GBS) method [12]. Details of SNP discovery and capture probe design and testing are described in [54]. The total number of SNPs markers genotyped was 1,371,123. The allele frequencies of these SNPs were calculated using the 117 unrelated individuals. Those SNP markers with a minor allele frequency of less than 0.03 were excluded from the analysis, leaving 67,168 SNP markers to be used in this study. The call rate of SNP markers for individual genotypes ranged from 0.60 to 0.93, with an average of 0.89. Where individual SNP genotypes were missing, substitution with the population mean for that SNP was used. Heterozygosity ranged from 0.11 to 0.35, with a mean of 0.28 and a standard deviation of 0.03 in POP2. Heterozygosity ranged from 0.11 to 0.41, with a mean of 0.27 and a standard deviation of 0.04 in POP3.

### Statistical models

In this study, the predictive ability of GEBVs estimated using a GBLUP model that was based on the genomic relationship matrix was compared with those estimated using an ABLUP model that was based on the average numerator relationship matrix. The genomic relationship matrix was calculated based on genomic information whereas the average numerator relationship matrix on pedigree information. This study aimed to demonstrate the efficacy of genomic selection for non-key traits in radiata pine using existing clonally replicated trial datasets. The genetic parameters and EBVs from ABLUP were estimated through the linear mixed model described in eq. (1), with the assumptions of homogeneous additive and non-additive genetic variances, heterogeneous residual variances, heterogeneous variances for replication, set within replication and incomplete block across sites. Attempts were made assuming heterogeneous genetic variance across all sites, but a full genetic variance-covariance matrix was unable to estimate due to small numbers of genotypes at some sites.

1 $$ y= X\beta +{Z}_aa+{Z}_dd+{Z}_rr+{Z}_ww+{Z}_bb+e $$

where *y* is a vector of measurements, *β* is a vector of fixed effects (intercept and site), *a* is a vector of polygenic additive genetic effects following $Var(a)\sim N\left(0,{\sigma}_a^2A\right)$ where ${\sigma}_a^2$ is the additive genetic variance and *A* is the pedigree-based average numerator relationship matrix [55], *d* is a vector of non-additive genetic effects following $Var(d)\sim N\left(0,{\sigma}_d^2I\right)$ where ${\sigma}_d^2$ is the non-additive genetic variance fitting both dominance and epistatic effects and *I* is the identity matrix, *r* is a vector of replication effects following *Var*(*r*)~*N*(0, *P*_0_ ⨂ *I*), where *P*_0_ is a replication variance-covariance structure matrix with $$ {P}_0=\left[\begin{array}{ccc}{\sigma}_{r_1}^2& \dots & 0\\ {}\vdots & \ddots & 0\\ {}0& 0& {\sigma}_{r_n}^2\end{array}\right] $$, ${\sigma}_{r_i}^2$ is the replication variance for site *i*, *w* is a vector of set nested within replication following *Var*(*w*)~*N*(0, *W*_0_ ⨂ *I*), where *W*_0_ is a set nested within replication variance-covariance structure matrix with $$ {W}_0=\left[\begin{array}{ccc}{\sigma}_{w_1}^2& \dots & 0\\ {}\vdots & \ddots & 0\\ {}0& 0& {\sigma}_{w_n}^2\end{array}\right] $$, ${\sigma}_{w_i}^2$ is the set nested within replication variance for site *i*, *b* is a vector of incomplete block effects following *Var*(*b*)~*N*(0, *B*_0_ ⨂ *I*) where *B*_0_ is a block variance-covariance structure matrix with $$ {B}_0=\left[\begin{array}{ccc}{\sigma}_{b_1}^2& \dots & 0\\ {}\vdots & \ddots & 0\\ {}0& 0& {\sigma}_{b_n}^2\end{array}\right], $$ ${\sigma}_{b_i}^2$ is the incomplete block variance for site *i*, *e* is a vector of residual effects following *Var*(*e*)~*N*(0, *R*_0_ ⨂ *I*) with $$ {R}_0=\left[\begin{array}{ccc}{\sigma}_{e_1}^2& \dots & 0\\ {}\vdots & \ddots & 0\\ {}0& 0& {\sigma}_{e_n}^2\end{array}\right] $$, ${\sigma}_{e_i}^2$ is the residual variance for site *i*, X, *Z*_*a*_, *Z*_*d*_, *Z*_*r*_, *Z*_*w*_ and *Z*_*b*_ are incidence matrices assigning fixed and random effects to measurements in vector *y*, and *n* is the number of sites.

The genetic parameters and GEBVs from GBLUP were estimated through the linear mixed model described in eq. (2), with the assumptions of homogeneous additive and non-additive genetic variances, heterogeneous residual variances, heterogeneous variances for replication, set within replication and incomplete block across sites:

2 $$ y= X\beta +{Z}_mm+{Z}_a{a}^{\ast }+{Z}_dd+{Z}_rr+{Z}_ww+{Z}_bb+e $$

where terms are the same as those in eq. (1) except *m* is the vector of genomic breeding values following $Var(m)\sim N\left(0,{\sigma}_m^2G\right)$, ${\sigma}_m^2$ is the additive genetic variance explained by markers and *G* is the normalized marker-based relationship matrix [56], a^*^ is the vector of residual polygenic additive genetic effects following $Var\left({a}^{\ast}\right)\sim N\left(0,{\sigma}_{a^{\ast}}^2A\right)$, where ${\sigma}_{a^{\ast}}^2$ is the additive genetic variance not explained by genetic markers, *Z*_*m*_ and ${Z}_{a^{\ast }}$ are the incidence matrices assigning random genomic effects and random residual additive genetic effects to measurements in vector *y*.

Three types of heritabilities were estimated and reported. Firstly, the pedigree-based heritability (${\hat{h}}_{a_i}^2\Big)$ was estimated on the basis of variance components obtained from the pedigree-based analysis (ABLUP) as ${\hat{h}}_{a_i}^2=\frac{{\hat{\sigma}}_a^2}{{\hat{\sigma}}_a^2+{\hat{\sigma}}_d^2+{\hat{\sigma}}_{e_i}^2}$, where ${\hat{\sigma}}_a^2$ is the additive genetic variance, ${\hat{\sigma}}_d^2$ is the non-additive genetic variance and ${\hat{\sigma}}_{e_i}^2$ is the environmental variance at site *i*. Secondly, marker-based heritability (${\hat{h}}_{m_i}^2\Big)$ representing the proportion of genetic variance explained by SNP markers in GBLUP, was estimated as ${\hat{h}}_{m_i}^2=\frac{{\hat{\sigma}}_m^2}{{\hat{\sigma}}_m^2+{\hat{\sigma}}_{a^{\ast}}^2+{\hat{\sigma}}_d^2+{\hat{\sigma}}_{e_i}^2}$, where ${\hat{\sigma}}_m^2$ is the additive genetic variance explained by SNP markers, ${\hat{\sigma}}_{a^{\ast}}^2$ is the residual additive genetic variance not explained by SNP markers but captured by pedigree, ${\hat{\sigma}}_d^2$ is the non-additive genetic variance and ${\hat{\sigma}}_{e_i}^2$ is the residual variance at site *i*. Finally, a combined marker-based and pedigree-based heritability (${\hat{h}}_{ma_i}^2$), representing the additive genetic variance fitted jointly by SNP markers and pedigree in GBLUP, was estimated as ${\hat{h}}_{ma_i}^2=\frac{{\hat{\sigma}}_m^2+{\hat{\sigma}}_{a^{\ast}}^2}{{\hat{\sigma}}_m^2+{\hat{\sigma}}_{a^{\ast}}^2+{\hat{\sigma}}_d^2+{\hat{\sigma}}_{e_i}^2}$**.**

The accuracy of both pedigree- and marker-based breeding values was estimated as $r=\sqrt{1-\frac{PEV}{\left(1+{F}_i\right){\hat{\sigma}}_a^2}}$[36], where *r* is the accuracy of breeding values, *PEV* is the prediction error variance, *F*_*i*_ is the inbreeding coefficient of individual genotype *i,* and ${\hat{\sigma}}_a^2$ is the estimated additive genetic variance linked to the breeding values, which is equal to ${\sigma}_a^2$ in ABLUP and to ${\sigma}_m^2$ in GBLUP. The percentage of additive genetic variance explained by SNP markers in GBLUP (%*VA*) was calculated as $\% VA=\frac{{\hat{\sigma}}_m^2}{{\hat{\sigma}}_m^2+{\hat{\sigma}}_{a^{\ast}}^2}\times 100\%$.

### Predictive ability and relative efficiency of genomic selection

The predictive ability of genomic selection refers to the accuracy achieved when GEBVs are used to predict the performance of individuals that are not included in the training population. Firstly, EBVs of all genotypes in the whole population were estimated with the ABLUP model using pedigree information and all phenotypes available. Secondly, GEBVs of all genotypes were estimated using the GBLUP model through a ten-fold random cross-validation procedure with ten replications [23]. The whole population was randomly divided into ten groups, each group having one-tenth of the genotypes. In each replication, GEBVs were estimated with the GBLUP model with nine groups of genotypes as a training population and the remaining group of genotypes as a validation population. Phenotypes of genotypes in the validation population were set as missing values. A Pearson product-moment correlation was calculated between GEBVs and EBVs of the genotypes in the validation population for each replication. The average of these correlations from ten replications was reported as the predictive ability of genomic selection.

The relative efficiency (*E*) of genomic selection over traditional BLUP selection that is based on phenotypes and pedigree indicates the benefit of genomic selection over traditional BLUP selection when generation interval is considered. Genomic selection is more efficient than traditional BLUP selection when *E* was over 100%, less efficient when *E* is below 100%, and equally efficient when *E* is equal to 100%. It was evaluated as a ratio of the expected genetic gain from GBLUP over the expected genetic gain from ABLUP per unit time. Therefore, we have:

$$ E=\frac{\frac{i{r}_{IH_g}{\sigma}_{A_g}}{L_g}}{\frac{i{r}_{IH_a}{\sigma}_{A_a}}{L_a}}\bullet 100\%=\frac{r_{IH_g{\sigma}_{A_g}}}{r_{IH_a}{\sigma}_{A_a}}\bullet \frac{L_a}{L_g}\bullet 100\% $$

where *i* is the selection intensity, ${r}_{IH_g}$ is the predictive ability of genomic selection that was calculated from 10-fold random cross-validation with GBLUP, ${r}_{IH_a}$ is the predictive ability of traditional BLUP selection that was calculated from 10-fold random cross-validation with ABLUP, ${\sigma}_{A_g}$ is the square root of additive genetic variance explained by SNP markers from GBLUP using all phenotypes, ${\sigma}_{A_a}$ is the square root additive genetic variance in ABLUP using all phenotypes, *L*_*g*_ is the generation interval of radiata pine for a selection based on GEBVs, and *L*_*a*_ is the generation interval of radiata pine for a selection based on EBVs. Based on a scenario analysis conducted by Li and Dungey [18] in conifers, *L*_*g*_ was 9 years in a forward selection strategy and *L*_*a*_ was 14 or 17 years for a forward selection with or without the establishment of a separate clonal archive of field-tested material, respectively.

### Comparison between two pedigrees

The documented pedigree of the training population was found with errors. The parentage of the training population was reconstructed using a panel of 704 SNPs with a call rate of greater than 0.75 and a minor allele frequency of 0.35 and 0.5. These parentage reconstruction SNPs did not deviate from Hardy-Weinberg equilibrium and did not show evidence of linkage disequilibrium between them. Details of selection criteria and parentage reconstruction performance of these SNPs were described in [54**].** An exclusion analysis approach that was described by Telfer et al. [57] was used to determine a trio relationship (a progeny and two candidate parents). For a given trio combination of a progeny and two candidate parents, an exclusion for the trio was considered if a SNP genotype in the progeny was not one of four genotypic combinations that were possibly formed from genotypes of the SNP in the candidate parents. The number of exclusions was calculated for all 704 SNPs for all possible trio relationship combinations of the documented candidate parents. Among all possible trio relationships related to a progeny, the trio relationship with the lowest number of exclusions was assigned as pedigree for the progeny.

## Data Availability

The data used in the manuscript are available without any restriction from the corresponding author on reasonable request.

## Abbreviations

ABLUP: Best linear unbiased prediction based on the average numerator relationship matrix
BLUP: Best linear unbiased prediction
EBV: Estimated breeding value
GBLUP: Best linear unbiased prediction based on genomic relationship matrix
GBS: Genotyping by sequencing
GEBV: Genomic estimated breeding value
GS: Genomic selection
KT: Key trait
NKT: Non-key trait
POP2 and POP3: Two training populations used in this study
RPBC: Radiata Pine Breeding Company
SNP: Single nucleotide polymorphism
